# Antibacterial peptide PMAP-37(F34-R), expressed in *Pichia pastoris*, is effective against pathogenic bacteria and preserves plums

**DOI:** 10.1186/s12934-023-02164-5

**Published:** 2023-08-27

**Authors:** Chunming Dong, Lijun Xu, Weitao Lu, Mengru Li, Rui Zhang, Yanyan Sun, Jian Liu, Xinlei Chu

**Affiliations:** 1 College of Marine and Environmental Sciences, Tianjin University of Science and Technology, Tianjin, 300457 China; 2 Department of Epidemiology and Biostatistics, Tianjin Medical University Cancer Institute and Hospital, Tianjin, 300060 China; 3 College of Agriculture and Bioengineering, Heze University, Heze, 274000 China; 4 Jinan Deheng Medical Technology Co., Ltd, Jinan, 250031 Shandong Province China

**Keywords:** Antimicrobial peptides, Antibacterial mechanism, Preservation

## Abstract

**Background:**

Recently, researchers have focused on the search for alternatives to conventional antibiotics. Antimicrobial peptides are small bioactive peptides that regulate immune activation and have antibacterial activity with a reduced risk of bacterial resistance. Porcine myeloid antibacterial peptide 37 (PMAP-37) is a small-molecule peptide with broad-spectrum antibacterial activity isolated from pig bone marrow, and PMAP-37(F34-R) is its analogue. In this study, PMAP-37(F34-R) was recombinantly expressed in *Pichia pastoris*, and the recombinant peptide was further investigated for its antibacterial properties, mechanism and preservative in plums.

**Results:**

To obtain a *Pichia pastoris* strain expressing PMAP-37(F34-R), we constructed a plasmid expressing recombinant PMAP-37(F34-R) (pPICZα-PMAP-37(F34-R)-A) and introduced it into *Pichia pastoris*. Finally, we obtained a highly active recombinant peptide, PMAP-37(F34-R), which inhibited the activity of both Gram-positive and Gram-negative bacteria. The minimum inhibitory concentration is 0.12–0.24 µg/mL, and it can destroy the integrity of the cell membrane, leading to cell lysis. It has good stability and is not easily affected by the external environment. Hemolysis experiments showed that 0.06 µg/mL-0.36 µg/mL PMAP-37(F34-R) had lower hemolysis ability to mammalian cells, and the hemolysis rate was below 1.5%. Additionally, 0.36 µg/mL PMAP-37(F34-R) showed a good preservative effect in plums. The decay and weight loss rates of the treated samples were significantly lower than those of the control group, and the respiratory intensity of the fruit was delayed in the experimental group.

**Conclusions:**

In this study, we constructed a recombinant *Pichia pastoris* strain, which is a promising candidate for extending the shelf life of fruits and has potential applications in the development of new preservatives.

**Graphical Abstract:**

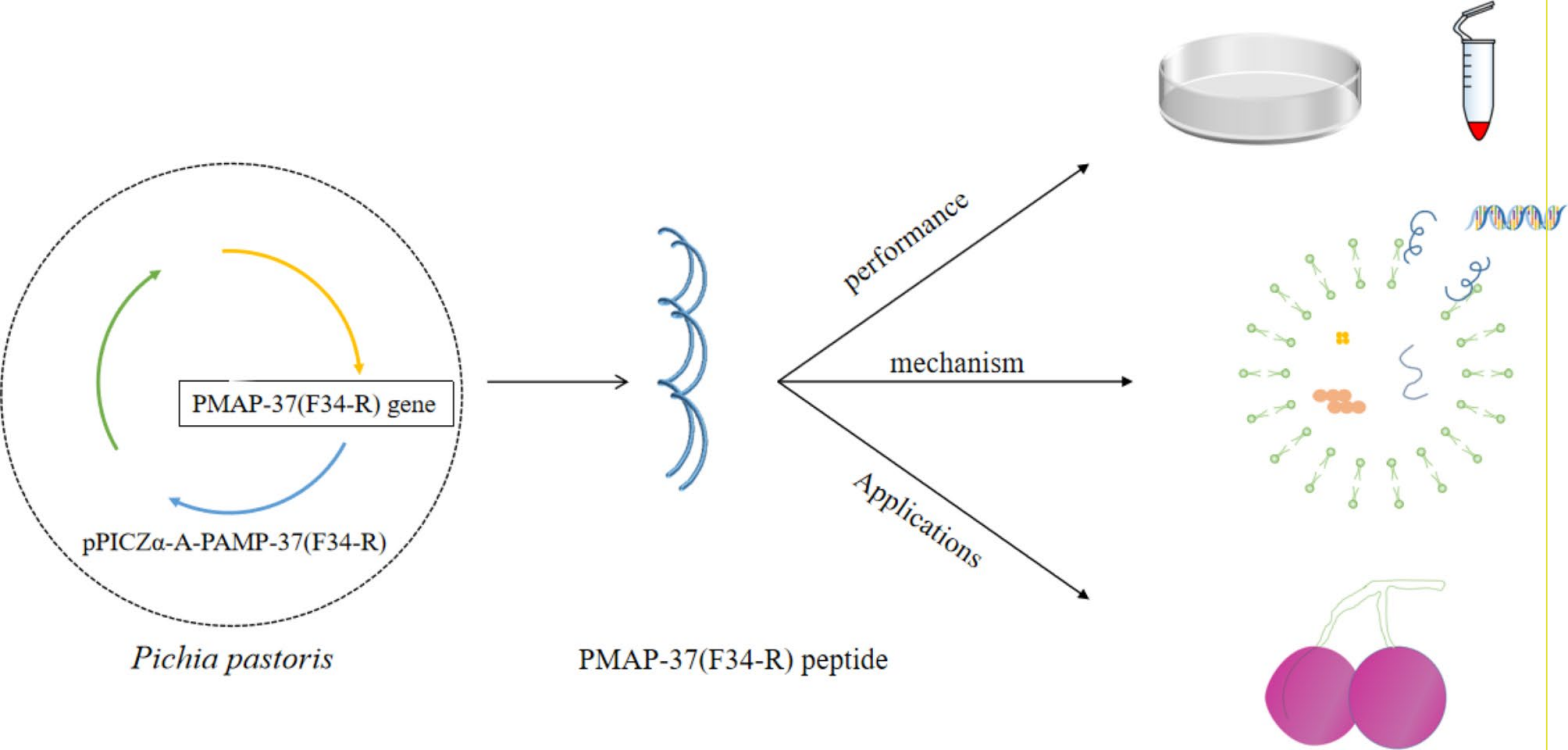

**Supplementary Information:**

The online version contains supplementary material available at 10.1186/s12934-023-02164-5.

## Introduction

The preservation of food has become a hot research topic because of its economic importance. Fruits are indispensable foodstuffs which are prone to spoilage. Such spoilage not only greatly affects human health, but also causes economic losses to producers and vendors. Plums are one of the best fruits in summer and are very popular with the public worldwide. However, plums exhibit strong respiration after being picked, and the ethylene content gradually increases, showing a typical ripening pattern of climacteric fruits [1, 2]. Withering is the main problem encountered when exporting plums, making it difficult to sell them. The transpiration of the plum results in reduced water distribution and fruit volume and folded skin [3, 4]. Second, long storage time and high temperature may accelerate the decay of the plums. Owing to the water loss and perishable nature of plums, their market value is limited [5, 6]. Consequently, it is difficult to maintain the desired state during road transportation and sales until they reach the consumer. Most conventional preservatives are chemically synthesized and can affect human health when consumed in excess. The application of safe preservative methods would not be detrimental to human and animal health and may not have a negative impact on the environment. Therefore, it is essential to develop novel green preservation methods.

Antimicrobial peptides are small-molecule active substances from various sources. It was first discovered in the insect immune system. Subsequently, similar peptides have been extracted from bacteria, fungi, amphibians, higher plants, and mammals. Such active peptides have a natural immune function and can act as an important molecular barrier for the host to resist the invasion of pathogenic microorganisms [7, 8]. Antimicrobial peptides have a wide antibacterial spectrum, low molecular weight, high thermal stability, and good water solubility. Current research has focused on molecular structure and improvement, mechanism of action, and other aspects [9]. Because antimicrobial peptides have a special mechanism of action, bacteria cannot easily develop resistance; therefore, they are likely to become new alternatives to conventional small-molecule antibiotics. Recently, the application of antimicrobial peptides has received increasing attention from researchers. Antimicrobial peptides from natural plants and animals are favored (because of their broad antimicrobial spectrum and low toxicity) and can be used as natural preservatives; for example, streptococcal lactate can kill heat-resistant bacilli and thus improve the shelf life of dairy products. In farming, antimicrobial peptides can also be used as food additives to prevent external infections and thus provide a good growing environment for livestock. In medicine, antimicrobial peptides can be used as a clinical treatment to reduce the harm caused by antibiotics. The research of antimicrobial peptides is becoming a hot spot and its market is expected to expand soon.

PMAP-37, a small cationic peptide composed of 37 amino acid residues, was isolated from the bone marrow of pigs. Compared with traditional antibiotics, PMAP-37 has broad-spectrum antibacterial activity and achieves bacterial inhibition by disrupting cell membranes but remains non-toxic to mammalian cells. Importantly, PMAP-37 exhibits low bacterial resistance. The analog of MAP-37, F34-R, is produced by substituting Arg for 34 Phe and has better activity than PMAP-37. Owing to the advantages of PMAP-37(F34-R), it has the potential to be used as a novel food preservative. However, traditional separation methods are unsuitable for mass production, owing to the low content of natural antimicrobial peptides, complex separation and purification steps, difficulty in extraction, and high cost [10]. Genetic engineering is often used to screen antimicrobial peptides with high expression, high antibacterial activity, and low toxicity, and further analyses are performed [11].

Currently, the expression of antimicrobial peptides by the *Pichia pastoris* system is a hot topic. First, the vector of *Pichia pastoris* contains an alcohol oxidase promoter, which can be regulated by methanol. Second, *Pichia pastoris* has a low nutritional requirement, low price, and high yield, and the produced antimicrobial peptide can be secreted into the extracellular space (unlike prokaryotic vectors, which require cell fragmentation). The secreted protein is mostly the target protein, which is easy to purify. Additionally, the genetic traits of *Pichia pastoris* are stable; the expression system does not easily lose genes and can modify the translated genes by glycosylation and protein phosphorylation, improving the bioactivity of the modified peptide. Such post-translational modification is difficult to achieve in a prokaryotic expression system.

In this study, we constructed a *Pichia pastoris* strain that efficiently expressed the recombinant peptide PMAP-37(F34-R). Subsequently, we determined the antibacterial properties and mechanism of PMAP-37(F34-R), including inhibitory activity against different Gram-positive and Gram-negative bacteria, minimum inhibitory concentration (MIC), stability, and hemolytic propensity. The integrity of the membrane was analyzed using propidium iodide (PI) staining and nucleotide leakage, and morphological changes of the membrane were analyzed using scanning electron microscopy. In this study, we aimed to evaluate the antibacterial activity and safety of PMAP-37(F34-R) to demonstrate its potential as a novel food preservative.

## Materials and methods

### Reagents, strains, and vectors

Experimental strains such as *Staphylococcus aureus* ATCC 25,923, *Escherichia coli* ATCC 10,305, *E. coli O157* ATCC 35,150, *Salmonella typhimurium* ATCC 10,467, *Listeria monocytogenes* ATCC21633, *Bacillus subtilis* ATCC6633, and XL10 were given by Nankai University. Strain GS115 was purchased from Poynter Bioengineering Co., and the pPICZαA-PMAP-37(F34-R) plasmid was purchased from GENEWIZ(CHINA). *Sac I* and *Rapid Taq Master Mix* were purchased from LABLEAD Inc. These materials have been used to construct genetically engineered bacteria and protein expression.

### Structure prediction

In order to predict the secondary structure of PMAP-37(F34-R), we used NPS online software (https://npsa-prabi.ibcp.fr/cgi-bin/npsa_automat.pl?page=npsa_sopma.html) for analysis and swiss-model (https://swissmodel.expasy.org/interactive) for peptide modeling. In addition, we used Pymol software to generate a surface view of the electrostatic potential of PMAP-37 (F34-R), and used Expasy software (https://www.expasy.org/resources/protscale) for hydrophobicity analysis.

### Strain construction and identification

We selected PMAP-37(F34-R) from the antimicrobial peptide database(https://dbaasp.org/search, ID: DBAASPS_18993). Next, we added six histidine tags. According to the codon preference of *Pichia pastoris*, the codon optimization of the whole gene sequence of PMAP-37(F34-R) was carried out, then the recombinant plasmid pPICZαA-PMAP-37(F34-R) was obtained. The PMAP-37(F34-R) target gene fragment was codon-optimized by adding EcoR I, ATG, 6×His, and “GAT GAT GAT AAG” at the N-terminal end, and 15 bases at the C-terminal end by adding the stop codons TAA and XbaI. The recombinant plasmid pPICZαA-PMAP-37(F34-R) was transformed into XL10 for amplification, linearized with *SacI* endonuclease and transformed into *Pichia pastoris* GS115 by heat shock method.

### Heat shock method and identification of positive strains

Linearized plasmids and heat shock solution were added to GS115 competent cells and then mixed well. 30 °C water bath for 30 min, halfway mixing; after adding dimethyl sulfoxide, the mixture was mixed well in 42 °C water bath for 15 min. The supernatant was discarded by instantaneous centrifugation at 12,000 rpm, and YPG was added to resuscitate at 30 °C for 1 h. The supernatant was discarded by instantaneous centrifugation at 12,000 rpm, resuspended with 100 µL 0.9% NaCl, coated the plate, picked up a single colony and activated it on a 100 µg/mL bleomycin-resistant plate, and then activated it on a 200 µg/mL resistance plate to increase the amount of gene copy [12].

The grown strain was amplified, and a polymerase chain reaction (PCR) was performed to verify whether the transformation was successful. PCR reaction conditions: Pre-denaturation at 94 °C for 5 min; denaturation at 94 °C for 1 min, annealing at 56 °C for 1 min, extension at 72 °C for 1 min, 35 cycles; extension at 72 °C for 10 min.

### Recombinant strains expressing antimicrobial peptides

Positive transformants of pPICZα-PMAP-37(F34-R)-A were selected for the induction of expression. Monoclonal clones were selected and cultured in 5 mL BMGY medium (1% yeast powder (m/v), 2% peptone (m/v), 10% glycerol (10%), 10% YNB (13.4%), 0.2% biotin (0.02%)). After 24 h, the clones were transferred to 25 mL of BMGY medium for amplification. The cells were cultured to OD_600_ = 6–8. The cells were collected through centrifugation and washed thrice with distilled water. The cells were transferred to BMMY medium (1% yeast powder (m/v), 2% peptone (m/v), 10% YNB (13.4%), 0.2% biotin (0.02%)). Subsequently, 1.5% methanol was added every day to induce expression. After fermentation, the supernatant was collected for bacteriostatic tests to verify whether the target product was active for subsequent bacteriostatic performance tests.

### Western blot and Elisa

The PVDF membrane was closed overnight at 4 °C in a refrigerator by adding 5% BSA blocking solution. Incubate with primary antibody (his-tag) and secondary antibody (HRP-Goat-anti-mouse) dilutions at room temperature for 2 h. Add luminescent solution for exposure. A standard curve was made according to the addition of HIS-tagged GFP protein, and the antimicrobial peptide pPICZαA-PMAP-37(F34-R) was added to the filter membrane in a 96-well plate. G250 staining solution was added to each well, and A595nm was measured by enzyme standardization within 3–5 min. pPICZαA-PMAP-37(F34-R) protein content could be derived from the standard curve.

### Inhibition zone and stability analysis

The inhibition zone method was performed as described in a previous study with slight modifications [13]. Fifty microliters of the bacterial solution was mixed with 15 mL lysogeny broth, and the inhibition zone of the six bacteria was determined by drilling after coagulation. The stability of PMAP-37(F34-R) against *S. aureus* was determined using the inhibition zone method. 4 µg/mL PMAP-37(F-34R) was dissolved in buffers with different pH (2, 4, 6, 8, and 10), different proteases (papain, pepsin, trypsin, and protease K), and different temperatures (4, 25, 37, 65, and 90 °C). The stability profiles under various pH, proteases, and temperatures were determined. Untreated buffer was used as a negative control.

### Determination of MIC

Determination of MIC was performed as described previously [14]. The antibacterial activity of PMAP-37(F34-R) was measured against *Staphylococcus aureus* ATCC 25,923, *Escherichia coli* ATCC 10,305, *E. coli O157* ATCC 35,150, *Salmonella typhimurium* ATCC 10,467, *Listeria monocytogenes* ATCC21633, and *B. subtilis* ATCC6633. PMAP-37(F34-R) was diluted to a series of gradient concentrations. The test bacteria were incubated until the OD_600_ was approximately 1.0, diluted 1000 times, and 100 µL of the bacterial solution was mixed with 20 µL of peptide solution in a 96-well plate; each test solution was repeated three times. LB medium (100 µL) plus replacement buffer solution (20 µL) was used as a negative control, and the OD_600_ value was measured after incubation of the 96-well plate at 37 °C for 12 h.

### Hemolytic activity assay

The hemolytic effect of antimicrobial peptides on human erythrocytes was determined as described by Liu [15]. Triton X-100 (1%) and phosphate-buffered saline (PBS) were used as positive and blank controls, respectively. First, blood cells were centrifuged at 150 × *g* for 10 min, the supernatant was removed, and the cells were washed three times with PBS. Subsequently, blood cells were diluted with PBS to 4% (v/v) and incubated with different concentrations of PMAP-37(F34-R) peptide at 37 °C for 1 h. After centrifugation at 150 × *g* for 10 min, the supernatant was transferred to a 96-well microplate and the absorbance values were measured at 576 nm using an enzyme marker.

### Cell membrane integrity and permeability experiments

The total nucleotide leakage experiment was performed according to a previously reported method with slight modifications [16]. *S. aureus* was collected, washed twice, and resuspended in PBS to an OD_600_ of 0.8. Antimicrobial peptides were added and incubated at 37 °C for various durations. The bacterial solution was centrifuged to remove *S. aureus*. The OD of the supernatant was recorded at a wavelength of 260 nm.

The permeability assay was performed as previously described with slight modifications [17]. *S. aureus* was until the OD_600_ reached 0.3–0.6, washed three times with PBS, and resuspended to 1 × 10^8^ CFU/mL. The bacteria (100 µL) were mixed with 50 µL of antimicrobial peptide and incubated at 37 °C for 30 min, with PBS as a control. The supernatant was centrifuged and discarded, the bacteria were resuspended with PI-diluted dye, and stained at 37 °C for 30 min. After washing three times with PBS, the unbound stain was removed, and 10 µL of PBS was taken; an appropriate amount of bacteriophage was added onto the slide, the cover glass was observed by fluorescence microscope.

### Morphological changes of bacteria after antimicrobial peptide treatment

This method was performed as previously described with slight modifications [18]. *S. aureus* and *Salmonella typhimurium* were incubated until the OD_600_ reached 0.5, washed three times with PBS, and resuspended. The PMAP-37(F34-R) concentration was 0.32 µg/mL. One hundred microliters of bacterial solution were collected, and 100 µL of fixative was added and fixed overnight at 4 °C. The fixative was aspirated, and 100 µL of 25, 50, 70, 80, 90, and 100% ethanol was added every 8 min in a sequential gradient of dehydration. Finally, the bacteria were suspended with 100% ethanol, placed on a coverslip, and air-dried for observation.

### Antiseptic effect of antimicrobial peptides on plums

Approximately 40 g of plums were used in the test, five in each group. The experimental group was sprayed with 5 mL of PMAP-37(F34-R) at a concentration of 0.36 µg/mL, while the control group was sprayed with 5 mL of sterile water, simulating hot weather from July to August, and placed at 30 °C to observe the decay, respiration, and weight loss.

### Statistical analysis

All results were subjected to one-way analysis of variance (ANOVA) and Duncan’s multiple range test using SPSS software. Results were defined as statistically different when P < 0.05.

## Results

### Structural analysis

**Fig. 1:**
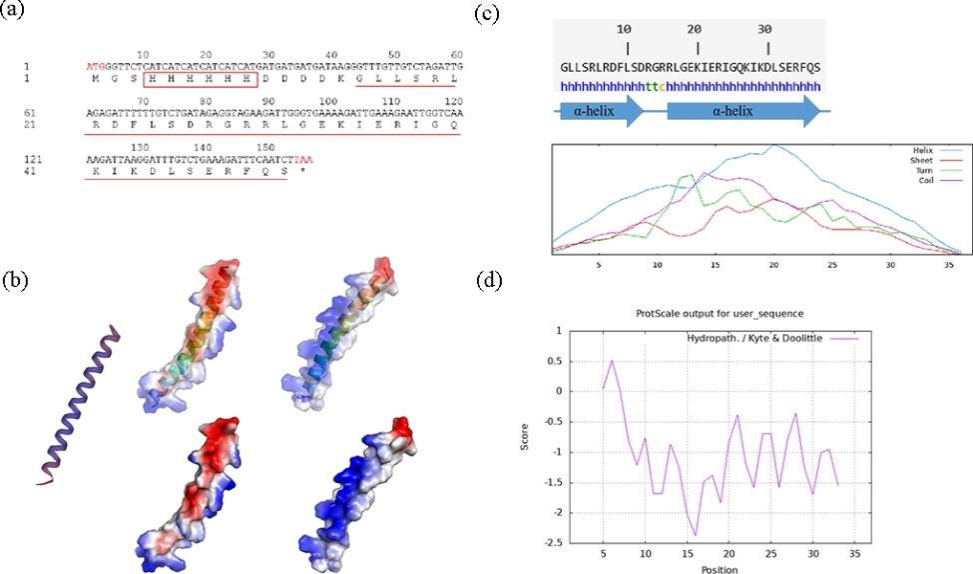
Sequence information of PMAP-37(F34-R) (**a**); secondary structure prediction (**b**); tertiary structure prediction and electrostatic potential analysis (**c**); hydrophobicity analysis (**d**)

The original and recombination sequences are shown in Table S1. The antimicrobial peptide PMAP-37(F34-R) is composed of 37 amino acids with a theoretical size of 4.374 kDa. Six histidine tags were added to the target gene design process to facilitate resolution (Fig. 1a). The secondary structure analysis showed that the α-helix structure in PMAP-37(F34-R) accounted for a relatively heavy proportion, which was an important factor in producing antibacterial ability (Fig. 1b). The tertiary structure showed that PMAP-37(F34-R) was a monomer structure. Further electrostatic potential analysis showed that most of the whole was positively charged, which was beneficial to the binding of PMAP-37(F34-R) to negatively charged cell membranes (Fig. 1c). Hydrophobic analysis showed that PMAP-37(F34-R) had an amphiphilic structure and was hydrophilic as a whole. Such a structure was more conducive to the role of antimicrobial peptides (Fig. 1d).

### Positive strain identification and target protein detection

**Fig. 2:**
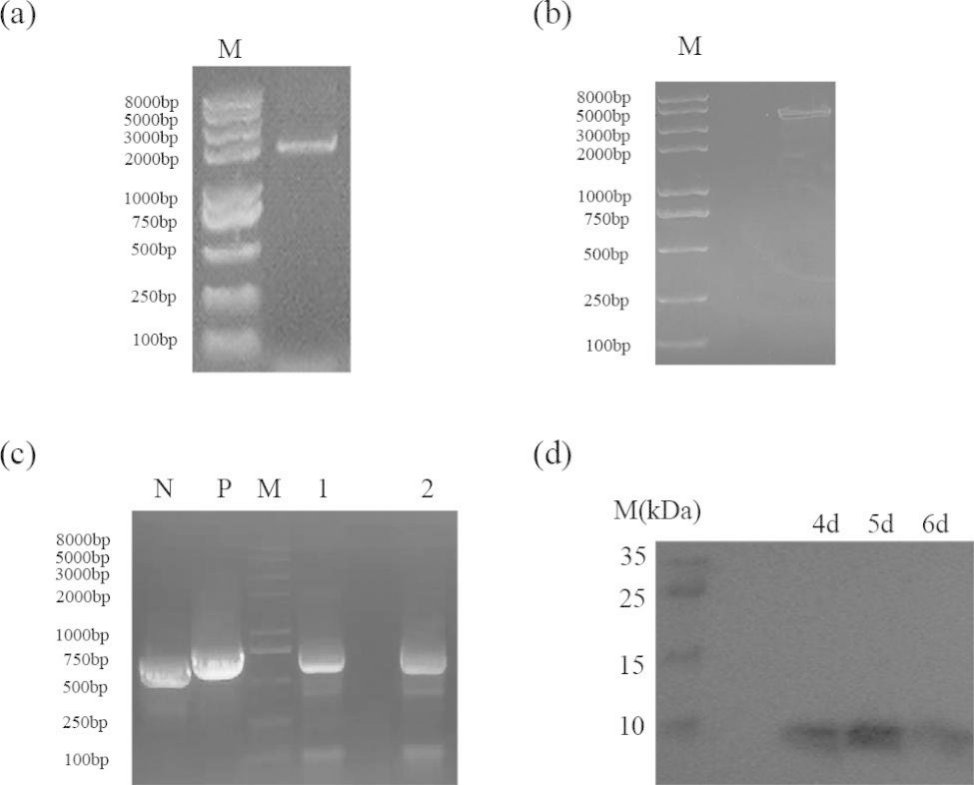
Plasmid extraction (**a**) and plasmid digestion verification (**b**); Positive clones were identified by PCR. The empty vector was used as negative control (N), and the vector containing target gene was used as positive control (**c**); The target protein was detected by Western blot

The plasmid map was shown in Fig. S1. The recombinant plasmid, pPICZαA-PMAP-37(F34-R) (Fig. 2a), was extracted, digested (Fig. 2b), and transferred into *Pichia pastoris*. The obtained strains were verified using PCR, primer sequence as shown in Table S2. The results are shown in Fig. 2c. N is the negative control empty vector, P is the positive control containing the target gene, and 1 and 2 are different *Pichia pastoris* transformants, indicating that the recombinant strain was successfully constructed. Strain 1 was selected and the supernatant was collected every 24 h after induction. Western blot results (Fig. 2d) showed that the target antimicrobial peptide was present in the supernatant and reached the highest concentration on the fifth day. To verify the antibacterial effect of the recombinant peptide PMAP-37(F34-R) against Gram-positive and Gram-negative bacteria, we performed antibacterial performance experiments.

### Antibacterial performance analysis

#### Determination of the inhibition circle and MIC

**Fig. 3:**
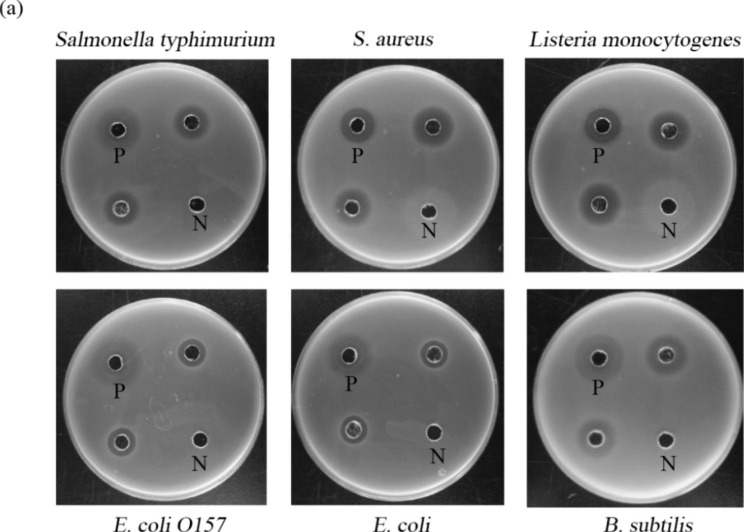
The antibacterial effect of PMAP-37(F34-R) on six bacteria strains were determined. P was 50 µg/mL gentamicin, N was the supernatant containing empty vector *Pichia pastoris* expression, and the rest were parallel samples (**a**) in the experimental group

The inhibitory effects are shown in Fig. 3. The positive control was 50 µg/mL gentamicin and the negative control was a supernatant solution containing the empty carrier *Pichia pastoris*. PMAP-37(F34-R) inhibited all six bacteria tested and was effective against three Gram-positive bacteria (*S. aureus*, *Listeria monocytogenes* and *B. subtilis*) and three Gram-negative bacteria (*Salmonella typhimurium*, *E. coli O157*, and *E. coli*). Based on this, the MIC of PMAP-37(F34-R) was determined.

**Fig. 4:**
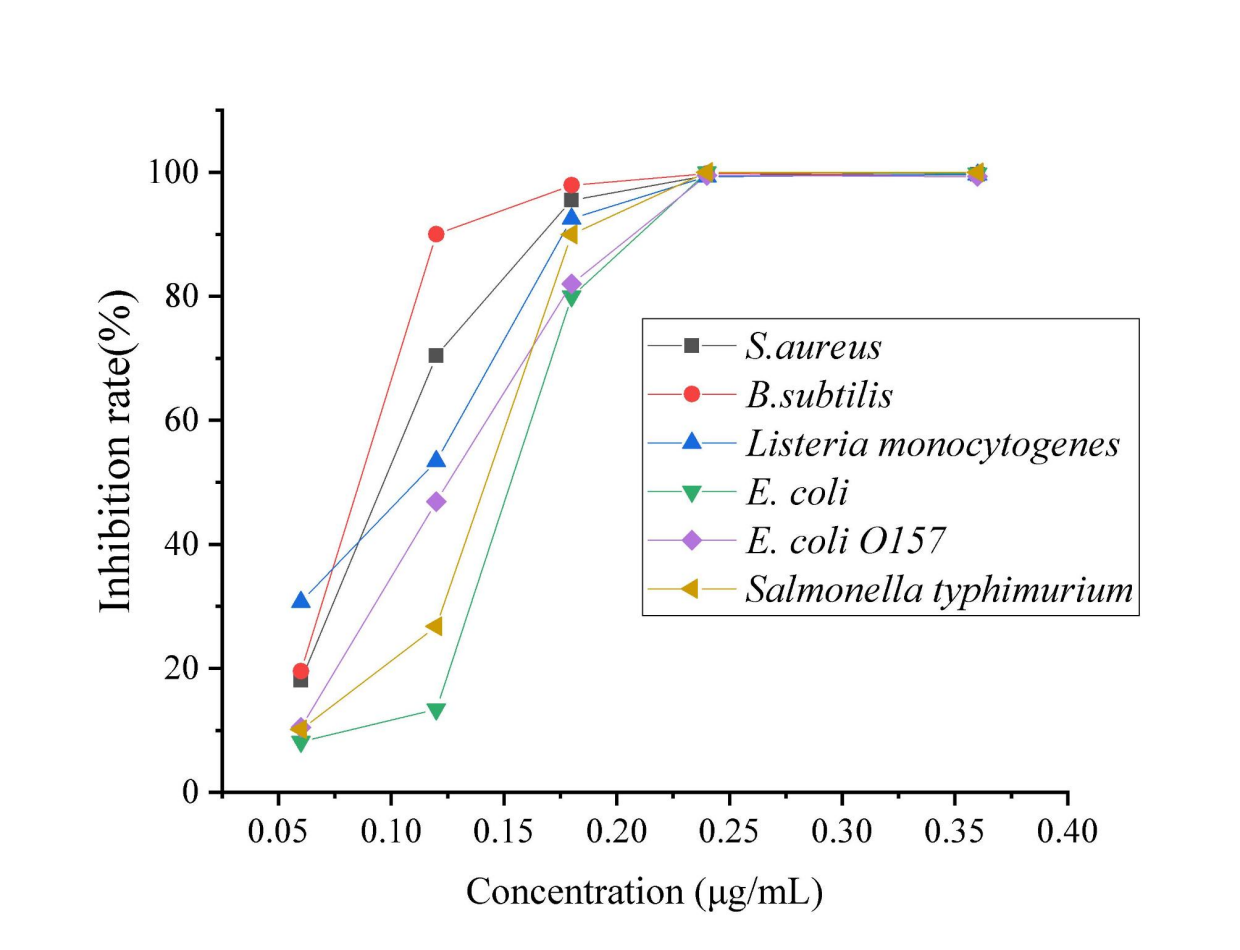
Determination of MIC

The MIC of PMAP-37(F34-R) was determined using six Gram-positive and Gram-negative bacterial strains. As shown in Fig. 4, PMAP-37(F34-R) had antibacterial effects on both bacteria, and the MICs varied between strains, ranging from 0.12 to 0.24 µg/mL (the inhibition rate reached more than 90%). Overall, PMAP-37(F34-R) was more effective against *S. aureus, B. subtilis*, *Salmonella typhimurium*, and *Listeria monocytogenes*.

#### Stability analysis

**Fig. 5:**
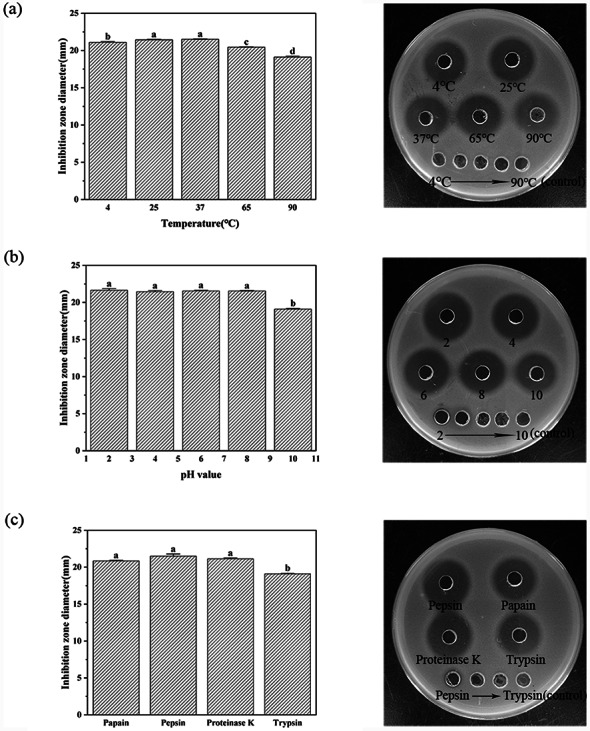
The stability of PMAP-37(F34-R) at different temperatures (**a**), pH (**b**) and protease (**c**)

The stability of PMAP-37(F34-R) was determined under harsh conditions including different temperatures, acids, bases, and protease treatments. As shown in Fig. 5, at different temperatures (Fig. 5a), pH (Fig. 5b), and protease treatment conditions (Fig. 5c), high temperature (90 °C), a strong base (pH = 10), and trypsin treatment partially affected the antimicrobial peptide, and otherwise maintained good stability. The results showed that the overall activity of PMAP-37(F34-R) was stable and not easy to inactivate under different temperatures, acid-base, and protease treatments.

#### Hemolytic activity assay

**Fig. 6:**
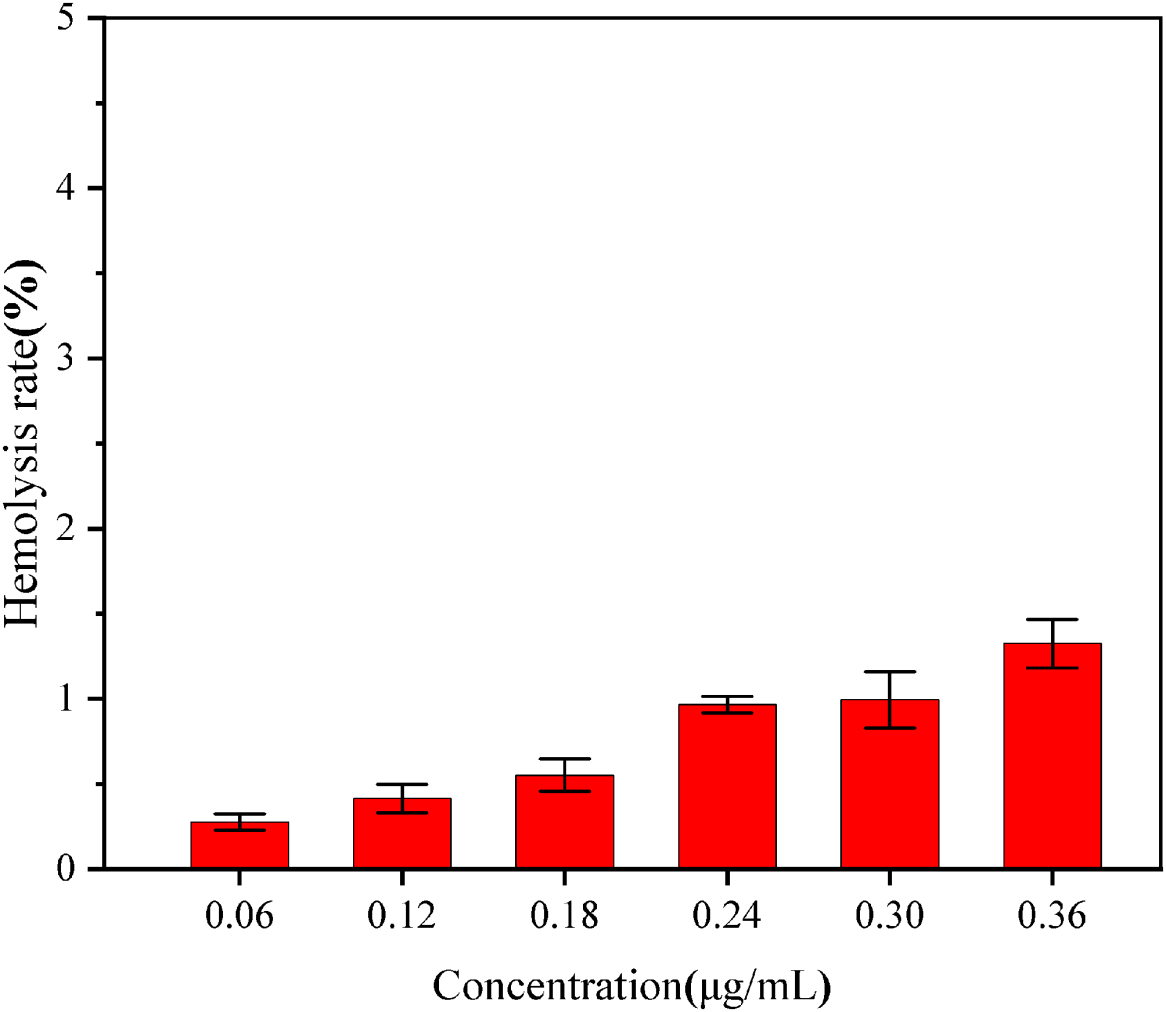
Hemolytic activity

According to the above results, it can be seen that the antimicrobial peptide PMAP-37(F34-R) has good antibacterial activity and good stability under harsh treatment conditions such as different temperatures, pH and proteases, so its potential application in the food field can be explored. However, if PMAP-37(F34-R) is applied to the food field, safety inspection is essential. The main purpose of hemolytic experiment is to detect whether antimicrobial peptides have an effect on human blood cells. Large hemolytic means that the substance will rupture red blood cells and is harmful to the human body. Therefore, our experiment aims to explore whether antimicrobial peptides are safe by hemolytic activity of human red blood cells. As shown in Fig. 6, when the concentration of PMAP-37(F34-R) was in the range of 0.06–0.36 µg/mL, the hemolysis rate of each experimental group was below 1.5%, indicating that PMAP-37(F34-R) at the concentration of 0.06–0.36 µg/mL had low hemolysis for human blood cells.

### Analysis of antibacterial mechanism

#### Cell membrane integrity and permeability experiments

**Fig. 7:**
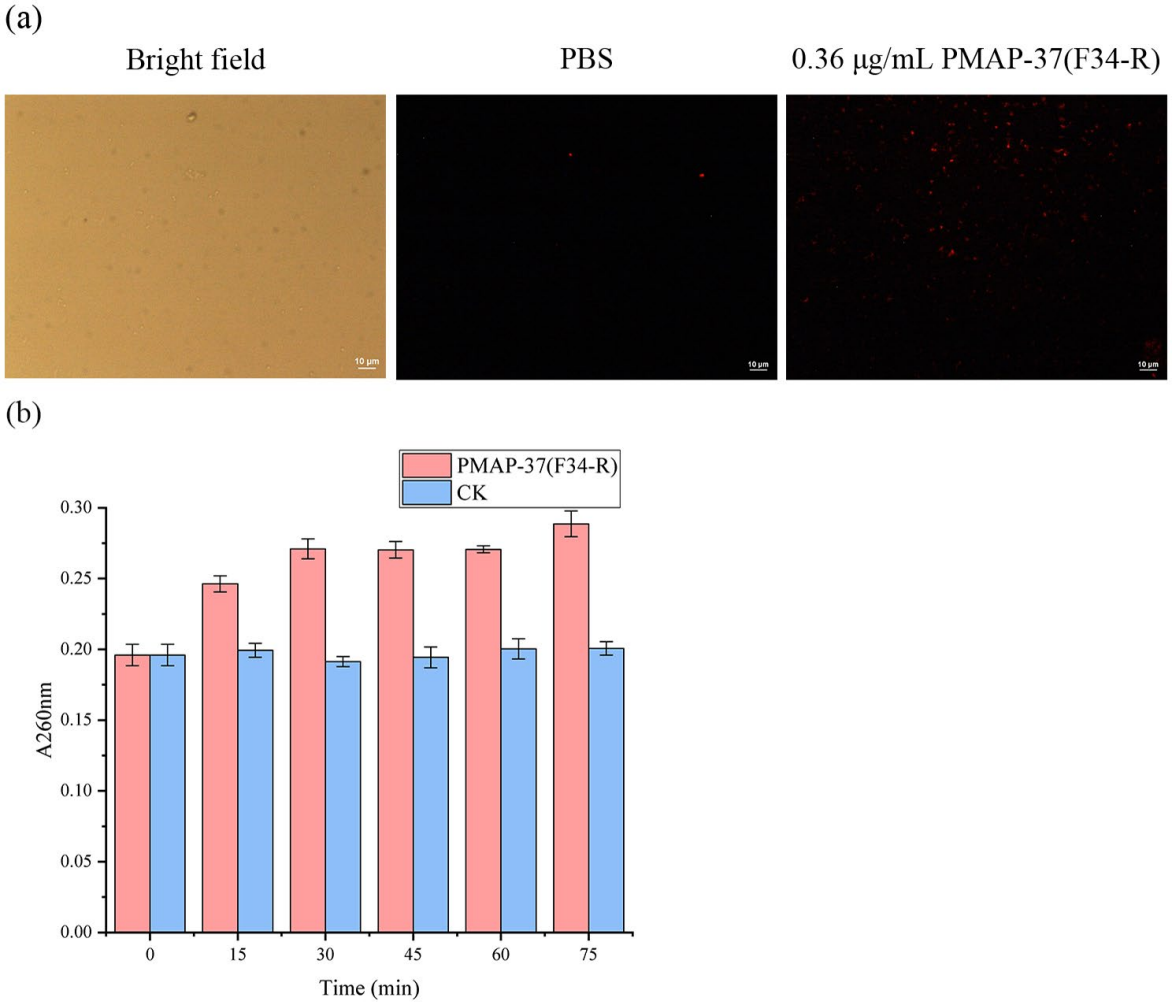
Membrane integrity (**a**) and nucleotide leakage (**b**)

Biofilms treated with antimicrobial peptide were used for PI staining analysis to confirm the killing effect of PMAP-37(F34-R) on biofilms. After antimicrobial peptide treatment, PI was able to penetrate the cell membrane and bind to bacterial nucleic acids, which produced red fluorescence under 488 nm excitation light, indicating impaired cell membrane integrity. Untreated control cells showed a small amount of red color, and extensive cell death (red color) was observed after the addition of PMAP-37(F34-R) (Fig. 7a). Therefore, PMAP-37(F34-R) can act on the cell membrane of bacteria, causing damage and, thus, an important cause of bacterial death. To further verify the integrity of the cell membrane, we measured total nucleotide leakage. The results showed (Fig. 7b) that nucleotide leakage increased significantly within 30 min after PMAP-37(F34-R) treatment, and that the rate of increase was more moderate. This indicates that PMAP-37(F34-R) disrupts the integrity of the cell membrane, leading to the leakage of the contents.

#### Analysis of morphological changes of bacteria after antimicrobial peptide treatment

**Fig. 8:**
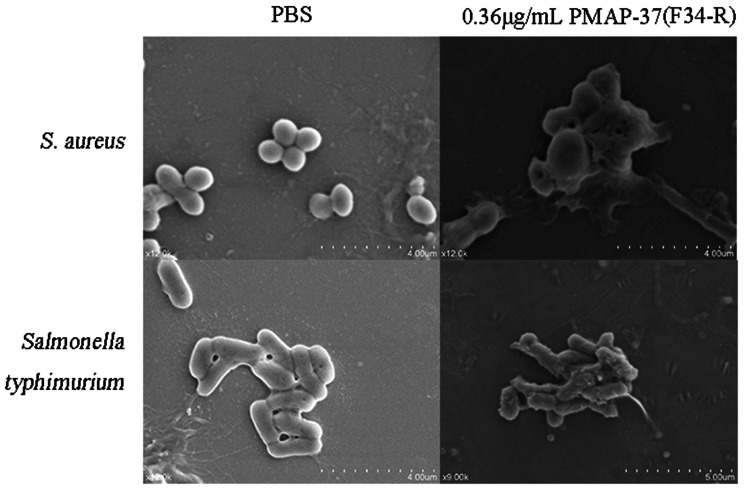
The changes of cell morphology were observed by scanning electron microscope

To further verify the antibacterial mechanism of PMAP-37(F34-R), we characterized the bacteria after treatment with an antimicrobial peptide. The cell morphology of *S. aureus* and *Salmonella typhimurium* after antimicrobial peptide treatment was observed using scanning electron microscopy (Fig. 8). The morphology of both bacteria treated with PBS was normal with smooth and intact cell membrane surface; the surface of *S. aureus* treated with PMAP-37(F34-R) was damaged with pore deformation, surface depression, and content leakage; the surface of *Salmonella typhimurium* treated with PMAP-37(F34-R) was crumpled and burr-like. The above results indicate that the interaction between the positively charged PMAP-37(F34-R) and the negatively charged cell membrane disrupts cell membrane integrity and leads to leakage of bacterial content.

### Analysis of the preservative effect of antimicrobial peptides on plums

#### Effect of PMAP-37(F34-R) on the decay rate of plums

**Fig. 9:**
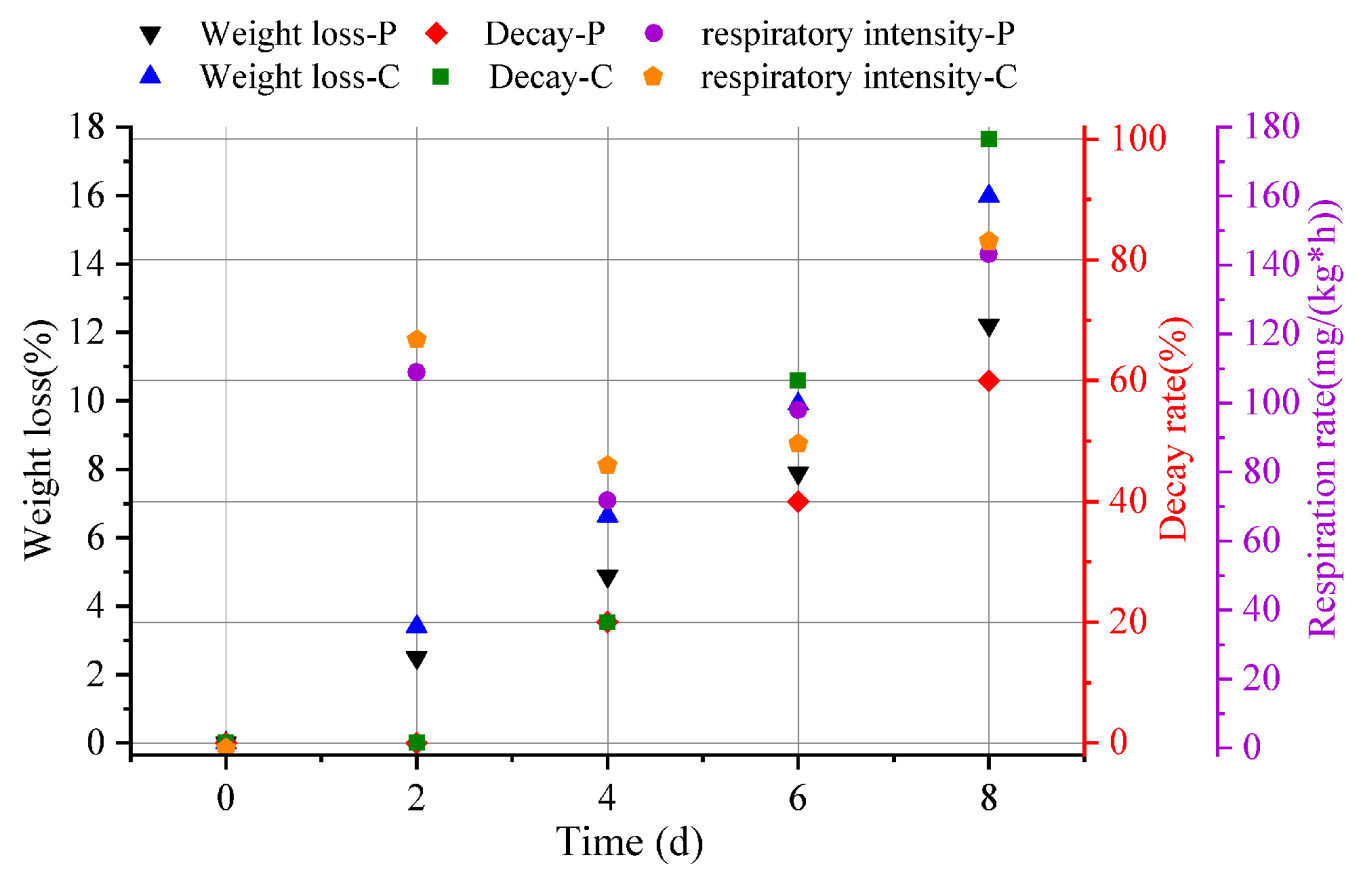
The changes of weight loss, decay and respiratory rate of plums were treated with PMAP-37(F34-R) (P), control group was sterile water (C)

The decay rate is the main appearance index used to determine the storage quality of fruits. As shown in Fig. 9, the decay rate of the two groups was equal on the fourth day of storage; after 4 d, the decay rate of the control fruit was accelerated, and the decay rate of the control fruit was 100% at 8 d of storage, while the decay rate of the experimental group treated fruit was 60%. The results showed that PMAP-37(F34-R) significantly inhibited the decay of plums under high-temperature conditions in the summer and had a certain preservation effect.

#### Effect of PMAP-37(F34-R) on the weight loss rate of plums

Transpiration on the surface of the fruit after picking leads to water and weight loss. The weight loss rate of the plum fruits treated with PMAP-37(F34-R) during storage is shown in Fig. 9. The weight loss rate of fruits in each treatment gradually increased with the extension of the storage period, and the weight loss rate of the control group reached 15.99% at 8 d of storage, while the weight loss rate of 0.36 µg/mL PMAP-37(F34-R)-treated group was 12.22%, which was significantly lower than the control. The results showed that PMAP-37(F34-R) significantly reduced the weight loss of plums under high-temperature conditions in summer and reduced the loss of nutrients in plums to a certain extent.

#### Effect of PMAP-37(F34-R) on the respiratory intensity of plums

Plum is a respiratory leap-type fruit that enters the respiratory leap period rapidly after picking, with a respiratory peak, followed by senescence and decay. Figure 9 shows that in the first 4 d, the respiratory intensity of the control and experimental groups decreased, and the respiratory intensity of the blank group was higher than that of the control group. On the sixth day, the respiratory intensity in the experimental group showed a significant increasing trend. On the eighth day, the respiratory intensity in the control group increased and was higher than that in the experimental group. Overall, the respiratory intensity of the experimental group was lower than that of the control group most of the time, indicating that the PMAP-37(F34-R) antimicrobial peptide reduced the respiratory intensity of fruits and vegetables, which might be related to the weight loss rate.

## Discussion

In this study, we showed that the peptide PMAP-37(F34-R) of *Pichia pastoris* prepared using DNA recombinant technology exhibited a wide range of antibacterial activities, including Gram-positive (*S. aureus*, *Listeria monocytogenes*, and *B. subtilis*) and Gram-negative (*E. coli O157*, *E. coli*, and *Salmonella typhimurium*) bacteria, with MIC values ranging from 0.12 to 0.24 µg/mL. In addition, when simulating extreme environmental conditions, the antibacterial activity of PMAP-37(F34-R) was partially affected by a high temperature of 90 °C, strong alkali (pH = 10), and trypsin treatment, while maintaining good stability in other cases. According to the results of the hemolysis assay, there were no significant differences between the groups of human blood cells treated with PMAP-37(F34-R) at 0.06 µg/mL to 0.36 µg/mL. In a study on the mechanism of bacterial inhibition by PMAP-37(F34-R), PI showed enhanced bacterial staining for antimicrobial peptide treatment, as well as increased nucleotide leakage, indicating that cell membrane integrity was disrupted. This was further confirmed by the results of scanning electron microscopy, where the cell membrane surfaces of *S. aureus* and *Salmonella typhimurium* treated with PMAP-37(F34-R) showed pores or folds, leading to leakage of contents, which resulted in cell death. Finally, in the study of the preservative effect of PMAP-37(F34-R) on plums, the decay and weight loss rates of the experimental group were significantly lower than those of the control group, and the respiratory intensity was also delayed. These results suggest that PMAP-37(F34-R) can significantly inhibit plum decay and is a promising food preservative. To date, we have only conducted experiments on fruit preservation. In other applications, PMAP-37(F34-R) also showed potential utilization value. In clinical application, by modifying the peptide segment, changing the primary and secondary structure of the antimicrobial peptide, increasing the positive charge of the antimicrobial peptide, prolonging the α-helix to increase the hydrophobicity, so as to enhance the antibacterial activity of the antimicrobial peptide [1, 19]. In addition, amino acids can be substituted to enhance the hydrophobicity of antimicrobial peptides, thereby reducing the probability of organisms being infected by pathogenic bacteria [20]. In animal experiments, the efficacy of PMAP-37(F34-R) was comparable to that of ceftifol sodium, and even exceeded that of antibiotics. Therefore, PMAP-37(F34-R) has been identified as a potential drug for the treatment of bacterial infections [21]. At present, there are many clinical application studies on PMAP-37(F34-R). The potential value of PMAP-37(F34-R) in manufacturing, animal husbandry and agriculture can be further explored, so as to bring certain benefits to social and economic development. Therefore, the fixed-point modification of PMAP-37(F34-R) and the exploration of its performance after modification will be the focus of our next research.

### Electronic supplementary material

Below is the link to the electronic supplementary material.

Supplementary Material 1: The plasmid map of pPICZ?A-PMAP-37(F34-R)

## Data Availability

All data generated or analyzed during this study are included in this published article.
