# Supplementary material for: Antibacterial peptide PMAP-37(F34-R), expressed in Pichia pastoris, is effective against pathogenic bacteria and preserves plums
Source: Microb Cell Fact. 2023 Aug 27;22:164. doi: 10.1186/s12934-023-02164-5 (PMC10464103; doi:10.1186/s12934-023-02164-5)

**Table S1.** The sequence of peptide

| Peptide | Sequence |
| --- | --- |
| PMAP-37(F34-R) | GLLSRLRDFLSDRGRRLGEKIERIGQKIKDLSERFQS |
| Recombination PMAP-37(F34-R) | GSHHHHHHDDDDKGLLSRLRDFLSDRGRRLGEKIERIGQKIKDLSERFQS |

**Table S2.** Primer sequence

| Primer | Sequence(5'to3') |
| --- | --- |
| pPICZaA-F | GAAGCTGTCATCGGTTACTCA |
| pPICZaA-R | TCCGCACAAACGAAGGTC |

**Fig. S1.** The plasmid map of pPICZαA-PMAP-37(F34-R)


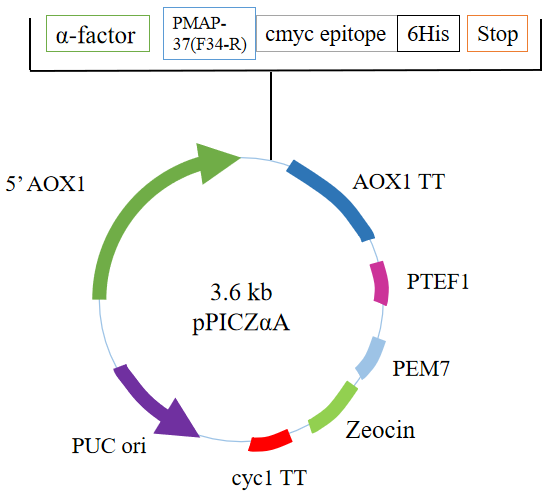

Supplement: Supplementary file 1 — Supplementary Material 1: The plasmid map of pPICZ?A-PMAP-37(F34-R) [file 12934_2023_2164_MOESM1_ESM.docx]
